# Prognostic Significance of the Microbiome and Stromal Cells Phenotype in Esophagus Squamous Cell Carcinoma

**DOI:** 10.3390/biomedicines9070743

**Published:** 2021-06-28

**Authors:** Olga Kovaleva, Polina Podlesnaya, Madina Rashidova, Daria Samoilova, Anatoly Petrenko, Valeria Mochalnikova, Vladimir Kataev, Yuri Khlopko, Andrey Plotnikov, Alexei Gratchev

**Affiliations:** 1 N.N. Blokhin National Medical Research Center of Oncology, 115478 Moscow, Russia; ovkovaleva@gmail.com (O.K.); polina.pod@yandex.ru (P.P.); madina.211@mail.ru (M.R.); dashasam@mail.ru (D.S.); epigenome@inbox.ru (A.P.); mochalnikova70@yandex.ru (V.M.); 2 Institute for Cellular and Intracellular Symbiosis, The Ural Branch of the Russian Academy of Sciences, 46000 Orenburg, Russia; vladimir0334@yandex.ru (V.K.); 140374@mail.ru (Y.K.); protoz@mail.ru (A.P.)

**Keywords:** cancer, stroma, macrophage, microbiome, esophagus, prognosis

## Abstract

Esophageal cancer is one of the most aggressive malignant neoplasms, with low survival rates and limited treatment options. In this study we analyzed the microbiome composition and the phenotype of inflammatory tumor infiltrate in squamous cell carcinoma of esophagus (ESCC) and examined possible relationships between them and their prognostic significance. We found that the predominant phyla of microorganisms found in both tumors and adjacent normal tissues were *Firmicutes, Proteobacteria, Actinobacteria, Gemmatimonadetes* and *Bacteroidetes*. We established that only bacteria of the genus *Staphylococcus* differ between tumors and normal tissues. We found a significant correlation between bacterial burden and the phenotype of the tumor stroma. Namely, a group of tumors characterized by a high expression of CD206 (r = −0.3976, *p* = 0.0056) in the stroma and iNOS (r = −0.2953, *p* = 0.0439) in tumor cells is characterized by a higher bacterial burden. Further, we established that in the group with a high content of CD206+ macrophages, there is also a predominance of gram-positive bacteria over gram-negative ones. We found that gram-positive bacterial burden is associated with disease prognosis in ESCC showing high content of CD206+ macrophages. In conclusion we established that the tumor microbiome, can be prognostically significant for ESCC when combined with other stromal markers.

## 1. Introduction

Esophageal cancer (EC) is one of the most aggressive malignant neoplasms with low survival rates and limited treatment options in the later stages. According to the International Agency for Research on Cancer (IARC), 572,000 new cases of EC were registered in the world in 2018 (3.2% of the total number of malignant neoplasms). In 2018, 508,600 deaths from EC were registered in the world (5.3% of the total number of deaths from cancer, ranking 6th) [1]. However, in most countries, including the Russian Federation, the situation remains critical. According to the statistics, the absolute number of newly diagnosed esophageal cancer cases in Russia increased from 2010 to 2015 by 10.4% in men and 2.1% in women, which indicates a pronounced gender difference in morbidity. In Western Europe and the United States, there is an annual 5% increase in esophageal cancer cases [2]. The most common morphological forms of EC are squamous cell carcinoma (95%) and adenocarcinoma (3%), whereas carcinosarcoma, small cell carcinoma, and melanoma are extremely rare.

Difficulties of EC treatment are due to late diagnosis explained by the largely asymptomatic course of the disease, as well as the high aggressiveness of the tumors. Notably, nearly 50% of patients with EC reveal distant metastases at the time of diagnosis [3]. In the treatment of EC, surgery, chemotherapy, radiotherapy, and immunotherapy are used. Targeted drugs include VEGFR2 antagonists, blocking the VEGF/VEGFR2 pathway and anti-Her2 antibodies, which suppress the proliferation of tumor cells. As well checkpoint inhibitors targeting the PD-1/PD-L1 interaction are used.

Investigation of tumor microbiome and its contribution to the development and progression of neoplasms attracts attention of researchers during the last decade. Considering the role of Helicobacter pylori in gastric cancer development [4], it can be assumed that the microbiome has a complex, multifactorial effect on the initiation and development of a malignant tumor, and its composition may be used as a prognostic factor.

Data on the microbiome of the healthy esophagus demonstrate its affinity to the oral microbiome [5]. Several studies describe differences in the esophageal microbiome of healthy individuals and patients with malignant esophageal lesions [6]. In particular, an increase in the proportion of *Enterobacteriaceae*, *Akkermansia* [6], *Lactobacillus fermentum* [7] and *Fusobacterium nucleatum* [8] in the microbiome of esophageal tumors has been noted. It is interesting to mark that the microbiome of adenocarcinoma of the esophagus has been studied quite well, while only few studies were done on the microbiome of squamous cell carcinoma of the esophagus.

In contrast to the microbiome, the phenotype of the stromal cells of esophageal tumors and its prognostic value have been studied quite well. Tumor-infiltrating CD8+ lymphocytes are associated with a favorable prognosis for both squamous cell carcinoma and esophageal adenocarcinoma [9]. Myeloid-derived suppressor cells (MDSC) and fibroblasts located in the tumor stroma suppress CD8+ T cells and may reduce the effectiveness of PD-1 blocking in both types of esophageal cancer, thereby affecting the effectiveness of immunotherapy [10,11]. The number of FoxP3+ T cells in the stroma of esophageal adenocarcinoma correlates with later stage and poor response to therapy [12,13], while a large amount of FoxP3 in the stroma of ESCC correlates with a good prognosis [14]. For the analysis of tumor associated macrophages several markers are used. General macrophage marker CD68 can be used to enumerate the total macrophage amount, while M1-specific (iNOS, IDO1 or HLA-DR) and M2-specific (CD163, CD204 or CD206) are used to identify the M2/M1 ratio. The number of CD68+ tumor associated macrophages does not correlate with the prognosis of patients with EC, while high numbers of CD163+ macrophages in the tumor stroma may be associated with both low survival, later stage, and the presence of metastases [15]. At the same time high numbers of CD163+ macrophages can be a favorable predictive factor [14]. Type 2 macrophages (M2) also correlate with high MMP9 expression and microvascular density in esophageal squamous cell carcinoma [16]. Cytotoxic M1 macrophages are associated with a favorable prognosis. Cao W. et al. showed that overall survival of patients with esophageal adenocarcinoma was inversely correlated with the M2/M1 ratio of macrophages [17]. In general, for tumors of the gastrointestinal tract, the prognostic significance of various stromal immune cells differs from that for other types of tumors. This is probably due to the fact that a large number of microorganisms normally inhabit the organs of the gastrointestinal tract, which can affect the phenotype of cells of the tumor microenvironment.

In this study we present a comprehensive analysis of the microbiome composition in squamous cell carcinoma of esophagus and the phenotype of inflammatory tumor infiltrate, followed by search for possible relationships between them and the prognostic significance of the identified correlations for patient survival.

## 2. Materials and Methods

### 2.1. Ethics Statement

The samples were collected in accordance with the guidelines issued by the Ethics Committee of the N.N. Blokhin National Medical Research Center of Oncology. All patients gave written informed consent (available upon request). The study was performed in accordance with the principles outlined in the Declaration of Helsinki.

### 2.2. Study Population

A total of 48 surgically resected and formalin-fixed paraffin-embedded (FFPE) human ESCC tissues were collected from the Clinical Oncology department of N. N. Blokhin Russian Cancer Research Centre (Moscow, Russia). The patients consisted of 36 men and 12 women with age range of 43–79 years old and mean age of 61 years old, all had been diagnosed with ESCC. Diagnoses were verified by histopathology, and only samples containing 70–80% or more tumor cells were used in the studies. Matched controls were histologically confirmed to be normal epithelial cells. The tumor samples were characterized based on the TNM according to the International System of Classification of Tumors, according to the staging classification of the Union for International Cancer Control. All specimens were sectioned into 4 μm sections and subjected to conventional hematoxylin and eosin staining. A diagnosis of ESCC was confirmed by pathologist following the World Health Organization histological tumor classification criteria. There were 10 cases of well-differentiated ESCC, 28 cases of moderately differentiated ESCC, and 10 cases of poorly differentiated ESCC. There were 23 cases with lymph node metastasis, 25 cases in clinical stages I–II, and 23 cases in clinical stages III–IV (Table 1). The survival status of all patients was followed up by post contact until December 2019. Overall survival (OS) was defined as the interval between surgery and death or between surgery and the last follow-up for surviving patients. Among the 42 patients who were recruited, 30 (81.0%) died, and 12 (19.0%) remained alive during the follow-up period. Patients who lived less than 2 months from the date of surgery were excluded from the analysis.

### 2.3. Immunohistochemical Study

Four micrometer thick sections were deparaffinized, heated to 110 °C for 10 min for antigen retrieval in ethylenediaminetetraacetic acid (EDTA) buffer pH 9.0. After cooling, endogenous peroxidase quenching was blocked by 3% hydrogen peroxidase for 5 min in room temperature (RT). Then the slides were blocked with 5% FBS (RT) for 15 min and incubated for 1 h with primary antibodies: anti-iNOS (SAB5500152; Sigma-Aldrich, St. Louis, MO, USA, 1:150 dilution), anti-CD206 (Sigma-Aldrich, St. Louis, MO, USA, HPA004114), anti-CD204 (Sigma-Aldrich, HPA000272), anti-CD68 (Genemed, South San Francisco, CA, USA, 61–0184), anti-PU.1 (Clone 9G7; Cell Signaling Technology, Danvers, MA, USA), anti-CD163 (Clone 10D6; BIOCARE Medical, Pacheco, CA, USA), anti-CD8 (Genemed, 61–0124), anti-CD3 (Genemed, 61–0011), anti-PD-L1 (Clone E1L3N, Cell Signaling) and anti-FOXP3 (Cell Signaling #98377). Antibody was removed and 100 µL DAB (UltraVision Quanto Detection System HRP DAB, Thermo Fisher Scientific, Waltham, MA, USA) was added to each section. We performed counterstaining with hematoxylin and washed the sections in dH_2_O two times for 5 min each. After dehydration, the sections were mounted with coverslips.

To score the immunostaining results for macrophages (CD68, CD163, CD206, CD204, PU.1) and T-cells (CD3, CD8), we randomly selected five representative high-power microscopic fields (×400 magnification) of the tumor sample per section, counted the numbers of positively stained cells (Olympus, Tokyo, Japan). Necrotic areas were ignored. The mean percentages of stained cells were counted as 0 (negative), 1 (≤10%), 2 (11–50%), and 3 (>50%). FoxP3 expressions were evaluated according to the average number of positively-stained cells in five randomly and averagely selected 400× high-power fields (HPF) in each case: 0 (no positive cells), 1 (1–5 positive cells), 2 (6–25 positive cells) and 3 (>25 positive cells) per HPF. Samples with scores 0–1 for CD206, CD204, CD8 and FoxP3 were combined in a group with low expression and samples with scores 2–3 were combined in a group with high expression. For CD68, CD163, PU.1 and CD3 samples with scores 0, 1 and 2 were combined in a group with low expression and samples with a score of 3 represented a group with high expression [18,19].

For iNOS and PD-L1 immunohistochemical staining was scored in tumor cells. Tumor staining was classified as positive when clear cytoplasmic staining for iNOS and membrane staining for PD-L1 was present in ≥ 1% of tumor cells. Since there are no clinically accepted thresholds for iNOS and PD-L1 expression, the following cutoffs were used for this stain expression: 0–≤ 1%, 1–1–10%, 2–10–50%, 3 > 50% of tumor cells showing cytoplasmic positivity. For the analysis of survival samples with scores 2 and 3 were combined in one group.

### 2.4. Quantitative PCR (qPCR)

Quantitative real-time PCR was performed to assess the abundance of the 16S gene present in a subset of tumor tissue. Following primers were used: F16S (5′-GACTCCTACGGGAGGC-3′) as the forward primer and R16S (5′-GCGGCTGCTGGCAC-3′) as the reverse primer; GP probe [FAM]-CTgAYSSAgCAACgCCgCg-[BHQ1] for gram-positive classification and GN-probe [JOE]-CCTgAYSCAgCMATgCCgCg-[BHQ1] for gram-negative classification [PMID: 20702819]. The PCR program was as follows: 95 °C for 10 min, 40 cycles of 95 °C for 15 s, 60 °C for 60 s. A total of 100 ng of extracted DNA and 0.25 μM of each primer, 0.15 μM GN-probe and 0.05 μM GP-probe were added to 4 μL of the PCR mix-DFMasZGTaqMIX-2025 (with SybrGreen) (Dialat, Moscow, Russia) and DFMasCFTaqMIX-2025 (without SybrGreen) (Dialat, Moscow, Russia), and DNA-free water was added up to 20 μL total volume. All reactions were performed in triplicates. A negative control containing DNA-free water instead of DNA was used for each PCR run. The real-time qPCR data analysis was performed with the BioRad software (Bio-Rad Laboratories, Hercules, CA, USA) with manually set threshold. For the purposes of analysis, we estimated a number of cycles to cross threshold (Ct value) as a measure of 16s rRNA gene load and hence bacterial burden. A higher bacterial burden resulted in a lower number of cycles to cross threshold, that is, a lower Ct value [19,20].

### 2.5. 16S rRNA Gene Library Preparation and MiSeq Sequencing

DNA extraction from tissues was performed using DNA FFPE kit (Qiagen, Hilden, Germany) according to the manufacturer instruction for capturing bacterial DNA. The quality of the extracted DNA was assessed with electrophoresis in 1% agarose gel and a Nanodrop 8000 (Thermo Fisher Scientific, Waltham, MA, USA). The DNA concentration was quantified using a Qubit 4.0 Fluorometer (Life Technologies, Carlsbad, CA, USA) with dsDNA High Sensitivity Assay Kit (Life Technologies, Carlsbad, CA, USA).

Preparation of the DNA libraries was performed according to the Illumina protocol (Part #15044223, Rev. B.) with primers targeting the V3–V4 regions of the SSU ribosomal RNA (rRNA) gene, S-D-Bact-0341-b-S-17 (5′-CCTACGGGNGGCWGCAG-3′) as the forward primer and S-D-Bact-0785-a-A-21 (5′-GACTACHVGGGTATCTAATCC-3′) as the reverse primer [21]. The reaction mixture (10 µL) contained both primers, 0.1 µM each; 80 µM dNTPs; 0.2 U Q5 High-Fidelity DNA Polymerase (New England Biolabs, Ipswich, MA, USA). Following PCR program was used: 95 °C for 3 min, 40 cycles 95 °C for 30 s, 56 °C for 30 s, 72 °C for 30 s, final extension 72 °C for 5 min. For each reaction three replicates were amplified. Then the replicates were mixed together and cleaned up using Agencourt AMPure XP beads (Beckman Coulter, Brea, CA, USA). Paired-end 2 × 300 bp sequencing was performed on the MiSeq platform (Illumina, San Diego, CA, USA) with the Reagent Kit v.3 (Illumina, San Diego, CA, USA).

DNA libraries preparing, sequencing and bioinformatics treatment were performed in the Center of Shared Scientific Equipment “Persistence of microorganisms” of Institute for Cellular and Intracellular Symbiosis UrB RAS (Orenburg, Russia).

### 2.6. Bioinformatics Treatment

At the first stage, the raw reads obtained as a result of sequencing were evaluated with FastQC v. 0.11.7. Evaluation was necessary to determine the parameters of further processing and included an assessment of quality and length of reads, presence of adapter sequences. Paired-end reads were merged with a minimum overlap of 40 bp and a *p*-value of 0.0001 using PEAR v. 0.9.10 (PEAR. Available online: http://www.exelixis-lab.org/web/software/pear (accessed on 25 June 2021)) [22]. Adapter sequences were removed with Trimmomatic v 0.36 (www.usadellab.org/cms/?page=trimmomatic) [23]. After merging and adapters removal, the reads were re-evaluated with FastQC v. 0.11.7. Subsequent treatment of merged reads was conducted with Usearch v. 9.2.64 (drive5.com/usearch) [24] and included quality filtering (expected error or maxee less than 1.00) and amplicon size selection (420-bp minimal size). Evaluation of the filtering quality was carried out with FastQC v 0.11.7. The next stage included dereplication and clustering of the filtered reads. As a result of dereplication and clustering, operational taxonomic units (OTUs) were formed. Chimeric sequences were detected and removed using the UCHIME2 algorithm [25]. Final OTUs were aligned to the initial merged reads using global alignment (usearch_global tool) at 97% level of similarity. As a result of global alignment, number of merged reads corresponded to every OTU was estimated. Contaminant OTUs were identified and removed via the usearch_ublast command by matching the sequences of trial samples and negative control samples. The taxonomic classification of sequences was conducted using the RDP reference database (rdp.cme.msu.edu/index.jsp) [26]. For OTUs with taxonomic position estimated at low level of support (ab_score less than 0.7), taxonomy was determined using the NCBI database blast.ncbi.nlm.nih.gov. OTUs identified as host (human) were removed from the dataset.

#### Availability of Data

Raw sequence data and metadata are available at the NCBI Sequence Read Archive under accession numbers SRR14184995-14184984, BioProject PRJNA720010, BioSamples SAMN18630738-18630757.

### 2.7. Statistical Analyses

Diversity of microbiomes within samples (alpha diversity) was evaluated with the indices inverse Simpson and Shannon. Similarity of microbiomes between samples (beta diversity) was assessed using the Bray-Curtis distance. To visualize similarity of microbiomes between samples, Principal Coordinates Analysis (PCoA) was performed. Taxa that were significantly different between ESCC and normal tissues we identified with Microbiome Analyst [27], developed for microbiome statistics applications. Differences in the overall microbial composition between ESCC and adjacent normal tissues and other groups were assessed by Wilcoxon rank-sum or Mann-Whitney nonparametric test.

IHC statistical analysis was performed using GraphPad Prism ver. 9 by GraphPad Software (San Diego, CA, USA). The Spearman rank correlation coefficient was used to compare between groups to examine the association between immune marker expressions and clinicopathological characteristics and bacterial burden. Survival length was determined as a time period from the date of surgery to the date of death or the last clinical attendance. Survival curves were derived using the Kaplan–Meier method, and differences between curves were analyzed using the log-rank test. In all analyses, *p* values ≤ 0.05 were considered statistically significant.

## 3. Results

### 3.1. Clinical Samples

This study included 48 patients operated for ESCC at the N.N. Blokhin National Medical Research Center of Oncology. All samples were paired, that is, they consisted of histologically verified tumor tissue and a sample of conditionally normal esophageal tissue of the same patient located as far as possible from the tumor. In the study we included esophageal squamous cell cancer only. Other histological types of malignant esophageal tumors were not included. Clinical characteristics of the 48 patients are presented in Table 1. The mean age of the patients was 61.3 ± 8.4 years.

### 3.2. Characterization of Esophageal Bacterial Communities

To analyze the composition of the microbial community, the 16S rRNA gene was sequenced in 10 pairs of DNA samples from ESCC tumor and corresponding adjacent normal tissue samples. Information on analyzed samples is presented in Table 2.

Analysis of the taxonomic composition of the microbial community of esophageal tissues revealed the presence of seven dominant phyla, 164 genera and 393 species. For further analysis, we took into account the genera of bacteria with an abundance level of more than 0.1%. There were 71 such dominant genera in normal tissues and 106 in tumors.

**Figure 1:**
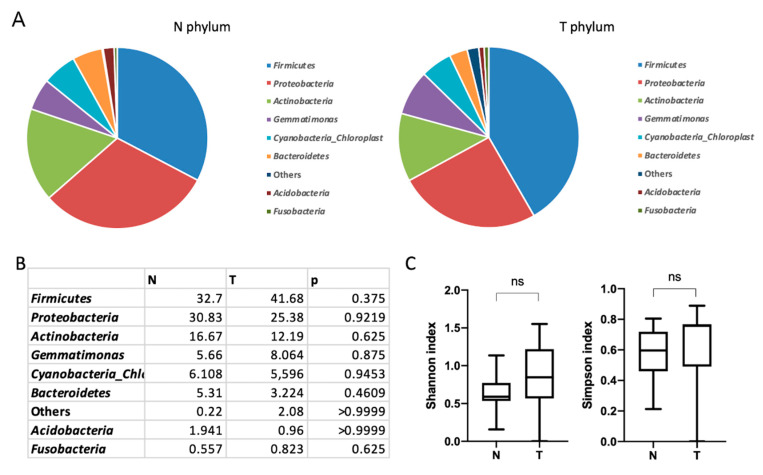
Taxonomic composition of the microbial community of esophageal tissues on the level of phylum. (**A**) Characterization of esophageal squamous cell cancer (ESCC) microbiota. Relative abundance at the phylum level for tumor (T) and normal tissue (N) samples. (**B**) Taxonomic composition. (**C**) Taxonomic α–diversity calculated with Shannon and Simpson indices between N and T groups. ns—non significant.

The predominant phyla of microorganisms found in both tumors and conventionally normal tissue samples were *Firmicutes, Proteobacteria, Actinobacteria, Gemmatimonadetes* and *Bacteroidetes* (Figure 1). There were no significant differences in the relative abundance of the microorganisms at the phylum level between tumor and adjacent normal tissues (Figure 1B). No significant differences for taxonomic alpha diversity were observed between tumors and normal adjacent tissues (Shannon and Simpson indices) at the phylum level (Figure 1C).

Next, the analysis of the relative abundance of bacteria at the genus levels in the tumors and adjacent normal tissues was performed.

**Figure 2:**
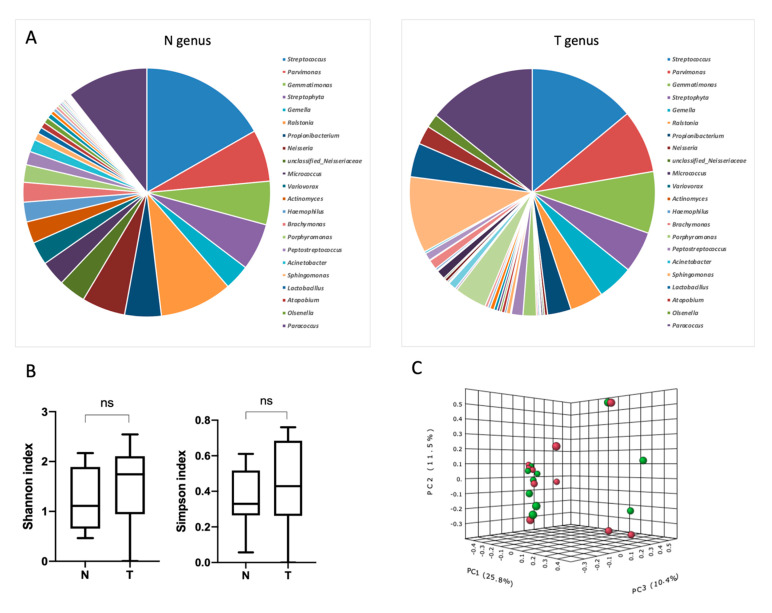
Taxonomic composition of the microbial community of esophageal tissues on the level of genera. (**A**) Characterization of ESSC microbiota. Relative abundance at the genus level for tumor and normal tissue samples. (**B**) Taxonomic α-diversity calculated with Shannon index and Simpson indices between N and T groups. (**C**) PCoA plot based on Bray-Curtis distance of NSCLC microbiome between tumor and normal tissues (red–N, green–T) [PERMANOVA] F–value: 0.50161; R–squared: 0.027111; *p*–value < 0.974.

The most represented genera of microorganisms found in tumor and normal tissue of the esophagus were the genera *Streptococcus, Parvimonas, Gemmatimonas, Ralstonia, Propionibacterium*. No significant differences for taxonomic alpha diversity were observed between the tumors and the normal adjacent tissues (Shannon and Simpson indices). To evaluate the similarities between all samples, the distances calculated on the basis of the unweighted UniFrac metrics, were visualized by PCoA plot. There was no significant distinct separation between the tumor and normal adjacent tissue groups at the genera level (Figure 2), with the exception of the genus *Staphylococcus*. The relative abundance of bacteria of the genus *Staphylococcus* in the tumor tissue was higher compared to the adjacent normal tissue (Supplementary Materials Table S1). Since bacteria of the genus *Staphylococcus* appeared to be the only genus that differed in their relative abundance between the groups of tumor and normal tissue, this genus was analysed at the species level. Two species of bacteria of this genus were found in the studied samples, namely *Staphylococcus pasteuri* and *Staphylococcus warneri*.

### 3.3. Gram+ and Gram− Bacteria Relative Abundance

**Figure 3:**
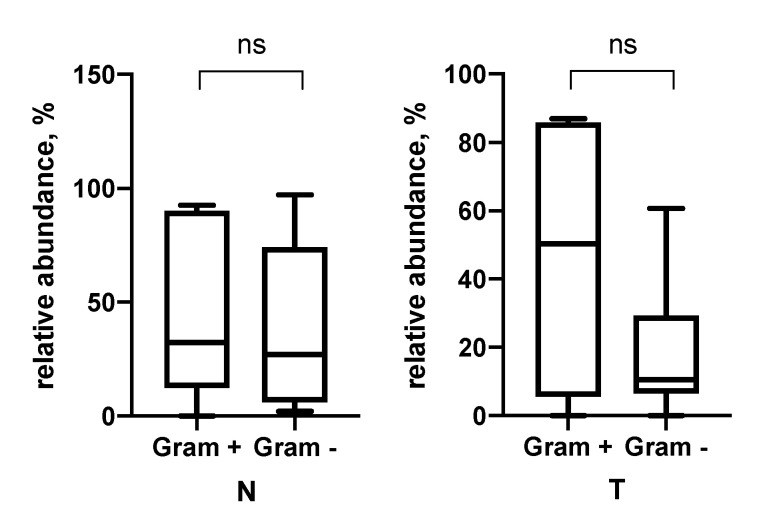
The relative content of Gram–positive and Gram–negative bacteria in the conditionally normal and tumor tissue of the esophagus.

Next, we analyzed the relative abundances of gram-positive and gram-negative bacteria in the tumor and normal tissue of the esophagus according to the sequencing results (Figure 3). It was found that in normal tissues approximately the same number of gram-positive and gram-negative bacteria is observed, while in tumors there is a tendency to a decrease in the content of gram-negative microorganisms.

### 3.4. Alpha-Diversity Depends on Tumor Stroma Phenotype

Next, we analyzed the alpha diversity of the esophageal microbiome depending on the phenotype of the tumor stroma (Table 3). We found no statistically significant differences in the alpha diversity of the microbiome in groups with a high and low content of macrophages (both M1 and M2), however, in groups of tumors with a high content of macrophages, in general, there is a tendency towards a decrease in these indicators.

When analyzing the inflammatory infiltrate from the side of T cells, we showed that the groups with a high and low content of cytotoxic T cells differ in their alpha diversity, namely, in the group with a high content of CD8+ cells, a significant decrease in the diversity index is observed.

The presence of CD8+ T-cells in tumors is inversely correlated with the content of bacteria of the genus *Staphylococcus* (r = −0.784, *p* = 0.011). The relative number of bacteria of this genus decreases significantly in this group. We hypothesize that this entails a change in the Shannon index, since a decrease in this index indicates a decrease in diversity and an increase in the dominance of certain genera. To confirm the increase in the dominance of individual genera, we also calculated the Simpson index. It was also significantly reduced in the group of tumors with high CD8+ T-cell infiltration.

### 3.5. Correlation and Survival Analysis of Bacterial Burden and Stroma

Next, we performed a quantitative analysis of the total bacterial burden, as well as Gram-positive and Gram-negative microorganisms in tumors, depending on their phenotype and clinical and morphological characteristics. The analysis of the bacterial content was done on the entire sample of 48 specimens by the real-time PCR. For statistical analysis, Spearman’s rank correlation coefficient was used. No association between the bacterial burden and the clinical and morphological characteristics of esophageal tumors was found (Table 4 and Supplementary Materials Tables S2 and S3). Further, a correlation analysis of the total bacterial burden with the phenotype of the tumor stroma was carried out.

As can be seen from the presented results (Table 5), a significant association between the level of bacterial burden and the phenotype of the tumor stroma is observed for CD206 (r = −0.3976, *p* = 0.0056) and iNOS (r = −0.2953, *p* = 0.0439).

**Figure 4:**
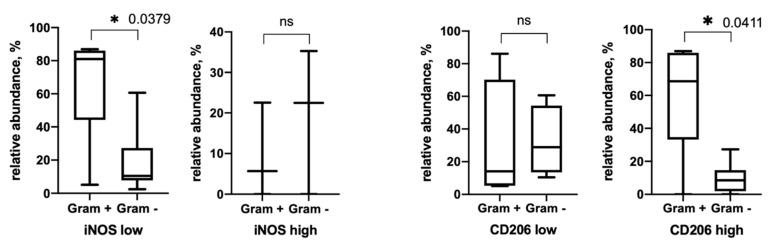
Relative abundances of Gram–positive and Gram–negative bacteria depending on the expression of iNOS and CD206 positive cells in esophageal tumors. * *p* < 0.05.

Namely, a group of tumors characterized by a high content of CD206 in the stroma and iNOS in tumor cells is characterized by a higher bacterial burden in general (Table 5). Further, we demonstrated that in the group with a high content of CD206 macrophages, there is a predominance of Gram-positive bacteria over gram-negative ones (Figure 4). The opposite situation is observed for iNOS. Namely, in the group of tumors with low iNOS expression, there is a predominance of Gram-positive bacteria over Gram-negative ones.

### 3.6. Survival Analysis

**Figure 5:**
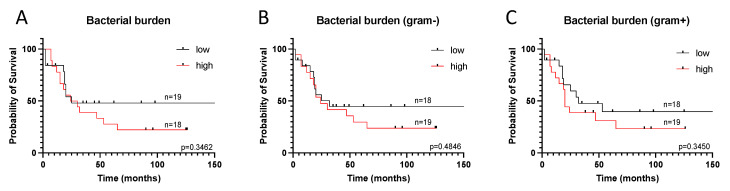
Survival of patients with ESCC versus total bacterial burden (**A**), Gram-negative bacterial burden (**B**) and Gram-positive bacterial burden (**C**). The differences were not statistically significant.

Next, we analyzed the survival of the patients depending on the total bacterial burden in tumors, the content of Gram-positive and Gram-negative bacteria, as well as in combinations with stromal markers using the construction of Kaplan-Meier survival curves. The results are presented in Figure 5.

As can be seen from the graphs presented, the total bacterial burden is not a prognostically significant parameter for ESCC. Next, we analyzed the survival rate depending on the total bacterial burden and the phenotype of the tumor stroma. We have previously shown that FoxP3 is a favorable prognostic marker for esophageal cancer [14]. Therefore, we analyzed FoxP3 together with the bacterial burden and found that FoxP3 is a marker of a favorable prognosis in the group of tumors with a high bacterial burden (HR = 0.3534, *p* = 0.0441) (Supplementary Materials Figure S1). For other analyzed markers, no correlations were found between survival and the bacterial burden.

**Figure 6:**
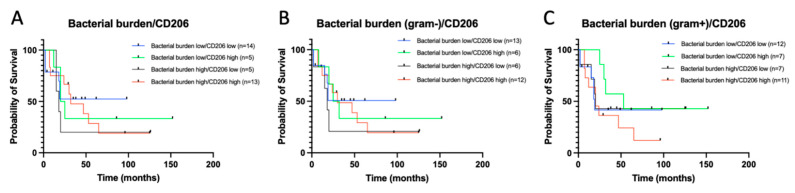
Survival of ESCC patients versus bacterial burden (**A**), Gram-negative bacterial burden (**B**) and Gram-positive bacterial burden (**C**) and CD206 + macrophages. No statistically significant differences were found for the groups shown on panels A and B. On the panel C the only statistically significant difference is between Bacterial burden high/CD206high and Bacterial burden low/CD206high groups (HR = 2.651, *p* = 0.044).

Previous analysis revealed a correlation between the CD206 content in tumors and the total bacterial burden [14]. However, the analysis of survival depending on the CD206 content, and the total bacterial burden did not reveal any patterns. We also showed that in tumors characterized by a high content of CD206, Gram-positive bacteria predominate, therefore we analyzed the survival in groups depending on the content of CD206 together with gram+ or gram- microorganisms (Figure 6).

Analysis of survival depending on the content of CD206 and gram-positive bacteria revealed poor prognosis in the case of combination of high CD206 and high Gram+ (HR = 2.651, *p* = 0.044) (Figure 6), while the most favorable prognosis was observed in the group with high CD206/low Gram+ (Figure 6). Median survival in the group bacterial burden (gram+) low/CD206 high was 53 months, while in bacterial burden (gram+) high/CD206 high it was 20 months. Obtained data indicate that bacterial burden can be used as a prognostic factor in the case of certain properties of tumor stromal cells. It has to be mentioned here that the sample size for this analysis was quite small, since survival data was only available for 42 patients and five patients were excluded, since they died within 2 months after surgery. Analysis of larger samples may reveal more correlations.

## 4. Discussion

EC is considered to be a quite common malignant neoplastic disease. Most patients die within a year after the diagnosis is made, as it is often detected at a later stage. There are two main subtypes of esophageal cancer-esophageal squamous cell carcinoma (ESCC) and esophageal adenocarcinoma (EAC), each with known risk factors and pathological features. ESCC accounts for up to 90% of esophageal cancers worldwide. Despite recent advances in diagnosis and therapy, the prognosis for esophageal cancer remains poor. Despite the fact that ESCC accounts for 90% of cases of this form of cancer, most of the microbiome research has mainly been conducted on adenocarcinomas. A very limited number of studies have been devoted to the microbiome of ESCC.

The microbiome of the esophagus plays an important role both in a healthy organism and in various pathologies. Previously, it was believed that the esophagus does not have its own resident microbiome due to its structure and functional load. Forty one genera of six main phyla have been found in esophagus, mainly *Firmicutes, Bacteroidetes, Actinobacteria, Proteobacteria, Fusobacteria*, and *TM7*. The most common genus was *Streptococcus* [28].

In a healthy organism, the esophageal microbiome is generally similar in composition to the oral microbiome and consists of six phyla: *Firmicutes; Bacteroidetes; Actinobacteria; Proteobacteria; Fusobacteria*; and *TM7* [5]. A distinctive feature of the esophageal microbiome is the absence of *Spirochaetes* [29]. It was found that representatives of the genus *Streptococcus* are the most prevalent in the microbiome of the healthy esophagus [30]. Snider et al. showed that there was no difference in microbial alpha diversity between the tissues of the normal esophagus and Barrett’s esophagus, but there was evidence of a decrease in diversity in EAC samples. They also found an increase in *Proteobacteria* along with a decrease in *Firmicutes* in high grade adenocarcinoma and dysplasia samples, and these tissues were characterized by an increase in *Enterobacteriaceae* and *Akkermansia* along with a decrease in *Veillonella* [6]. Elliott et al. found a predominance of *Lactobacillus fermentum* in EAC compared with tissue from healthy patients (*p* = 0.028) [7]. Li et al. demonstrated that in comparison with the control group, in the samples of ESCC, the number of *Fusobacteria* was higher (7.01 versus 1.66%, *p* = 0.039), and the number of *Actinobacteria* was lower (1.61 versus 4.04%). They also concluded that monitoring the microbiota of the esophagus may be an important method for predicting tumor recurrence after esophagostomy [31]. The role of *Fusobacterium nucleatum* as a promising prognostic biomarker for esophageal cancer was discussed by Yamamura et al., who found that higher amount of *F. nucleatum* in tumor tissue correlates with a poor prognosis and also activates CCL20, which promotes tumor progression [8]. Similar to *Helicobacter pylori*, *Campylobacter* spp. may play a role in the development of esophageal cancer through toxin-mediated inflammation [32]. However, at present, the microbiome associated with esophageal cancer is not fully understood.

In this study we analyzed the microbiome composition of ESCC tumors in comparison with normal tissues. We found that the predominant phyla of microorganisms found in both tumors and conventionally normal tissue samples were *Firmicutes; Proteobacteria; Actinobacteria; Gemmatimonadetes* and *Bacteroidetes;* which is consistent with available studies [33]. Like Wang et al., we showed no α diversity between the tumor group and normal esophageal tissue [33]. The lack of differences in alpha diversity between similar groups was also demonstrated by other researchers [34]. Further, after performing analysis at the level of genera, we showed that only bacteria of the genus *Staphylococcus* differ in the groups of tumors and normal tissues. Species analysis revealed that the bacteria of the genus *Staphylococcus* are represented by two species: *Staphylococcus warneri* and *Staphylococcus pasteuri*. The predominance of *Staphylococcus warneri* in tumors over normal tissue has been described for gastric cancer [35]. For esophageal cancer, no differences in the relative abundance of bacteria of these species have been previously described. The main limitation of this and similar studies is the usage of FFPE tissues for the isolation of DNA. However, as demonstrated in previous studies [19], this type of clinical material is suitable for metagenome sequencing.

Bacteria inhabiting tumors, on the one hand, can affect the tumor cells themselves by production of certain toxins or through certain oncogenic factors [36,37]. Also, they can influence the immune cells of the microenvironment [38]. Therefore, at the next stage of the work, we assessed the relationship between the microbiological and immunological components of esophageal tumors. We found a significant correlation between the level of bacterial burden and the phenotype of the tumor stroma. Namely, a group of tumors characterized by a high expression of CD206 (r = −0.3976, *p* = 0.0056) in the stroma and iNOS (r = −0.2953, *p* = 0.0439) in tumor cells is characterized by a higher bacterial burden as a whole. An interesting question is what causes such increase of bacterial burden? The first possible option is that a change in the composition of the tumor microbiome is due to the dominance of certain genera that leads to changes in the composition of the tumor microenvironment. It can be also hypothesized that qualitative and quantitative changes in the tumor microbiome are due to specific phenotype of the tumor stroma.

We have shown here that tumors generally have a greater number of gram-positive bacteria. Further, we established that in the group with a high content of CD206+ macrophages, there is also a predominance of gram-positive bacteria over gram-negative ones. Various studies show that gram-positive organisms are the leading cause of the invasive bacterial disease in patients with cancer. Immunosuppression induced by the underlying cancer or its attendant therapy synergize to make cancer patients particularly susceptible to Gram-positive infections. A broad range of Gram-positive bacteria cause serious infections in the cancer patient with the greatest burden of disease being due to staphylococci, streptococci, and enterococci [39].

Thus, for squamous cell carcinoma of the esophagus, two types of tumors can be identified, which significantly differ in their prognosis. Both types show high content of CD206+ macrophages, but differ in the content of Gram-positive bacteria. The first type is characterized by a high bacterial burden and has a poor prognosis. These tumors are dominated by gram-positive bacteria, which apparently determines their “immunosuppressive” phenotype. The predominance of gram-positive bacteria occurs due to a decrease in the relative abundance of Gram-negative microorganisms in this group of tumors (for example, the genus *Gemmatimonas*, r = −0.742, *p* = 0.033). The second type of tumors is characterized by a low gram-positive bacterial burden and favorable prognosis. This group of tumors is characterized by an association between the relative abundance of Gram-negative bacteria (r = −0.83, *p* = 0.013) and their total number in the tumor (r = −0.76, *p* = 0.029) and the amount of iNOS. Namely, the predominance of gram-negative bacteria in this type of tumor promotes an increase in iNOS expression and an active inflammatory response, which ultimately may contribute to a favorable prognosis. Thus, a detailed study of the tumor microenvironment and the composition of the microbiome moves researchers closer to real personalization in oncological practice.

In conclusion, the presented data indicate that the sole analysis of the tumor microbiome is not sufficient to evaluate its prognostic significance. We established that the tumor microbiome can be prognostically significant, when combined with the phenotype of tumor associated macrophages. Thus, a detailed study of the tumor microenvironment together with the composition of the microbiome moves researchers closer to real personalization in oncological practice.

## Figures and Tables

**Table 1:** Study population.

Category	All Cases
Age	
≤60	26 (54%)
>60	22 (46%)
Gender	
male	36 (75%)
female	12 (25%)
Stage	
I–II	25 (52%)
III–IV	23 (48%)
Nodal status	
N-	25 (52%)
N+	23 (48%)
Histologic grade	
G1/2	38 (79%)
G3	10 (21%)

**Table 2:** Clinicomorphololgic characteristics of samples used for sequencing.

#	Histology	TNM	Stage	Grade
1	SCC	T4N3M0	III	G3
2	SCC	T4N0M0	III	G2
3	SCC	T3N0M0	II	G2
4	SCC	T4NxMx	IV	G3
5	SCC	T3N0M0	II	G1
6	SCC	T1N0M0	I	G2
7	SCC	T2N2M0	III	G2
8	SCC	T3N0M0	II	G3
9	SCC	T3N1M0	III	G2
10	SCC	T2N0M0	II	G3

**Table 3:** Analysis of α-diversity depending on the phenotype of the inflammatory infiltrate.

	Shannon Index ± SD			Simpson Index ± SD		
	Low	High	*p*	Low	High	*p*
CD68	2029 ± 0.15	1084 ± 0.35	0.0556	0.7650 ± 0.03	0.4889 ± 0.14	0.1508
CD163	1674 ± 0.30	1282 ± 0.39	0.5167	0.6552 ± 0.11	0.5612 ± 0.10	0.5167
CD206	1493 ± 0.24	1599 ± 0.38	0.4762	0.6690 ± 0.08	0.5990 ± 0.13	0.7619
PU.1	1953 ± 0.14	0.962 ± 0.31	0.1143	0.7643 ± 0.03	0.4210 ± 0.15	0.1143
PD-L1	1717 ± 0.36	1316 ± 0.25	0.2571	0.6731 ± 0.11	0.5195 ± 0.06	0.1167
iNOS	1752 ± 0.24	1101 ± 0.39	0.1833	0.6783 ± 0.06	0.5073 ± 0.21	0.6667
CD3	1874 ± 0.09	1081 ± 0.31	0.2571	0.7434 ± 0.02	0.4524 ± 0.11	0.2571
CD8	1970 ± 0.12	0.593 ± 0.30	0.0167 *	0.7642 ± 0.02	0.3069 ± 0.15	0.0167 *
FOXP3	1584 ± 0.29	1447 ± 0.41	0.8889	0.6244 ± 0.10	0.6374 ± 0.11	0.5333

* Statistically significant.

**Table 4:** Correlation analysis of the total bacterial burden with clinical and morphological characteristics.

	Bacterial Burden vs. Age	Bacterial Burden vs. Gender	Bacterial Burden Vs. Grade	Bacterial Burden vs. Stage	Bacterial Burden vs. *n*
**Spearman r**					
r	0.03016	−0.01079	0.1418	0.09904	0.01886
95% confidence interval	−0.2674 to 0.3225	−0.3050 to 0.2853	−0.1600 to 0.4194	−0.2020 to 0.3830	−0.2778 to 0.3123
*p* value					
*p* (two-tailed)	0.8405	0.9426	0.3416	0.5078	0.8998

**Table 5:** Correlation analysis of the total bacterial burden with the phenotype of the tumor stroma.

	**Bacterial Burden vs. CD68**	**Bacterial Burden vs. CD163**	**Bacterial Burden vs. CD206**	**Bacterial Burden vs. CD204**	**Bacterial Burden vs. PU1**
**Spearman r**					
r	−0.08035	0.003585	−0.3976	−0.1854	−0.1761
95% confidence interval	−0.3668 to 0.2200	−0.2919 to 0.2984	−0.6200 to −0.1161	−0.4556 to 0.1162	−0.4480 to 0.1256
***p* value**					
*p* (two-tailed)	0.5914	0.9809	0.0056 *	0.2123	0.2365
	**Bacterial Burden vs. PD-L1**	**Bacterial Burden vs. iNOS**	**Bacterial Burden vs. CD3**	**Bacterial Burden vs. CD8**	**Bacterial Burden vs. FoxP3**
**Spearman r**					
r	0.2535	−0.2953	0.07555	0.09317	−0.08894
95% confidence interval	−0.04507 to 0.5104	−0.5431 to −0.0001028	−0.2246 to 0.3626	−0.2077 to 0.3779	−0.3743 to 0.2118
***p* value**					
*p* (two-tailed)	0.0856	0.0439 *	0.6138	0.5334	0.5522

* *p* < 0005.

## Data Availability

Unless otherwise stated in Materials and Methods sections data are available from the lab upon reasonable request.

## Supplementary Materials

The following are available online at https://www.mdpi.com/article/10.3390/biomedicines9070743/s1, Supplementary Table S1: Taxonomic composition by genus (%), Supplementary Table S2: Values of Spearman’s correlation coefficients (r), Supplementary Table S2: *p*-value, Supplementary Figure S1: Prognostic value of bacterial burden/FoxP3 positive cells ratio in ESCC.
