# Supplementary material for: Prognostic Significance of the Microbiome and Stromal Cells Phenotype in Esophagus Squamous Cell Carcinoma
Source: Biomedicines. 2021 Jun 28;9(7):743. doi: 10.3390/biomedicines9070743 (PMC8301468; doi:10.3390/biomedicines9070743)

Supplementary Data

Supplementary Table S1. Taxonomic composition by genus (%).

Genus	N	T	*p*
Streptococcus	16.8	14.0	>0.999
Parvimonas	6.7	8.3	0.438
Gemmatimonas	5.7	8.1	0.875
Streptophyta	6.1	5.4	0.945
Gemella	3.3	4.7	>0.999
Ralstonia	9.5	4.4	0.438
Propionibacterium	4.8	3.1	0.432
Neisseria	5.7	0.4	0.750
unclassified_Neisseriaceae	3.5	0.2	>0.999
Micrococcus	3.3	0.2	0.875
Variovorax	3.0	0.0	>0.999
Actinomyces	2.8	0.2	0.188
Haemophilus	2.6	0.2	0.813
Brachymonas	2.6	0.2	0.813
Porphyromonas	2.3	1.8	0.625
Peptostreptococcus	1.7	1.5	0.750
Acinetobacter	1.6	0.1	0.844
Sphingomonas	0.9	0.5	>0.999
Lactobacillus	0.8	0.2	0.625
Atopobium	0.8	0.4	0.250
Olsenella	0.7	0.3	0.500
Paracoccus	0.0	0.3	0.250
Solobacterium	0.6	0.4	0.250
Fusobacterium	0.5	0.5	0.875
Mogibacterium	0.5	0.3	0.625
Peptoniphilus	0.4	0.4	>0.999
Staphylococcus	0.3	4.2	0.039
Haematobacter	0.3	0.3	>0.999
Rothia	0.2	1.0	0.313
Bifidobacterium	0.2	0.2	0.750
Burkholderia	0.2	0.2	0.875
Halomonas	0.0	0.4	0.313
Oribacterium	0.2	0.2	0.625
Granulicatella	0.1	1.2	0.375
Selenomonas	0.1	0.1	>0.999
Slackia	0.1	0.1	0.500
Leptotrichia	0.1	0.3	0.625
Corynebacterium	0.1	1.3	0.125
Veillonella	0.1	0.1	0.875
Prevotella	0.1	1.0	0.438
Okibacterium	0.0	0.3	0.094
Comamonas	0.0	10.0	>0.999
Saccharopolyspora	0.0	4.4	0.500
Pantoea	0.0	2.5	>0.999
unclassified_Bacteria	0.0	1.8	0.500
**Others**	10.6	14.3	>0.999

Supplementary Table S2. Correlation analysis of bacterial burden and tumor stroma phenotype with clinicomorphological characteristics. (values of Spearman's correlation coefficients (r)).

	**Gender**	**Grade**	**Stage**	**N**	**CD68**	**CD163**	**CD206**	**CD204**	**PU1**	**PD-L1**	**iNOS**	**CD3**	**CD8**	**FoxP3**	**Bacterial burden**	**Bacterial burden (gram−)**	**Bacterial burden (gram+)**
**Gender**		−0.059	−0.072	−0.072	0.033	−0.057	0.194	0.046	0.082	0.242	−0.009	−0.108	0.102	0.281	−0.011	−0.189	0.013
**Grade**	−0.059		0.124	0.227	0.020	0.045	−0.229	−0.012	0.019	0.140	−0.151	0.100	0.071	−0.148	0.142	0.209	0.107
**Stage**	−0.072	0.124		0.750	0.015	0.056	0.051	−0.022	−0.142	−0.005	0.236	−0.164	−0.214	−0.060	0.099	0.280	0.077
**N**	−0.072	0.227	0.750		0.087	0.056	0.051	−0.024	−0.066	−0.080	0.216	−0.164	−0.054	−0.164	0.019	0.141	0.068
**CD68**	0.033	0.020	0.015	0.087		0.679	0.354	0.524	0.752	0.394	0.221	0.397	0.460	0.166	−0.080	−0.222	−0.092
**CD163**	−0.057	0.045	0.056	0.056	0.679		0.413	0.648	0.585	0.416	0.075	0.371	0.419	0.338	0.004	−0.033	0.031
**CD206**	0.194	−0.229	0.051	0.051	0.354	0.413		0.474	0.424	0.226	−0.028	0.087	0.055	0.450	−0.398	−0.181	−0.187
**CD204**	0.046	−0.012	−0.022	−0.024	0.524	0.648	0.474		0.604	0.154	0.255	0.372	0.241	0.293	−0.185	−0.126	−0.162
**PU1**	0.082	0.019	−0.142	−0.066	0.752	0.585	0.424	0.604		0.479	0.121	0.602	0.542	0.350	−0.176	−0.294	−0.122
**PD-L1**	0.242	0.140	−0.005	−0.080	0.394	0.416	0.226	0.154	0.479		−0.164	0.392	0.545	0.448	0.253	−0.100	−0.015
**iNOS**	−0.009	−0.151	0.236	0.216	0.221	0.075	−0.028	0.255	0.121	−0.164		−0.200	−0.149	−0.044	−0.295	−0.186	−0.079
**CD3**	−0.108	0.100	−0.164	−0.164	0.397	0.371	0.087	0.372	0.602	0.392	−0.200		0.735	0.341	0.076	−0.133	−0.134
**CD8**	0.102	0.071	−0.214	−0.054	0.460	0.419	0.055	0.241	0.542	0.545	−0.149	0.735		0.222	0.093	−0.187	−0.043
**FoxP3**	0.281	−0.148	−0.060	−0.164	0.166	0.338	0.450	0.293	0.350	0.448	−0.044	0.341	0.222		−0.089	−0.071	−0.050
**Bacterial burden**	−0.011	0.142	0.099	0.019	−0.080	0.004	−0.398	−0.185	−0.176	0.253	−0.295	0.076	0.093	−0.089		0.651	0.348
**Bacterial burden (Gram−)**	−0.189	0.209	0.280	0.141	−0.222	−0.033	−0.181	−0.126	−0.294	−0.100	−0.186	−0.133	−0.187	−0.071	0.651		0.505
**Bacterial burden (Gram+)**	0.013	0.107	0.077	0.068	−0.092	0.031	−0.187	−0.162	−0.122	−0.015	−0.079	−0.134	−0.043	−0.050	0.348	0.505	

Supplementary Table S3. Correlation analysis of bacterial burden and tumor stroma phenotype with clinicomorphological characteristics. (*p*-values of correlations presented in the supplementary table S2.)

	**Gender**	**Grade**	**Stage**	**N**	**CD68**	**CD163**	**CD206**	**CD204**	**PU1**	**PD-L1**	**iNOS**	**CD3**	**CD8**	**FoxP3**	**Bacterial burden**	**Bacterial burden (gram−)**	**Bacterial burden (gram+)**
**Gender**		0.689	0.626	0.626	0.826	0.699	0.186	0.755	0.580	0.098	0.951	0.464	0.492	0.053	0.943	0.204	0.933
**Grade**	0.689		0.401	0.121	0.890	0.762	0.117	0.936	0.900	0.344	0.305	0.500	0.632	0.316	0.342	0.159	0.473
**Stage**	0.626	0.401		0.000	0.919	0.704	0.729	0.880	0.336	0.975	0.106	0.266	0.145	0.685	0.508	0.057	0.607
**N**	0.626	0.121	0.000		0.558	0.704	0.729	0.871	0.656	0.587	0.140	0.266	0.714	0.265	0.900	0.343	0.652
**CD68**	0.826	0.890	0.919	0.558		0.000	0.013	0.000	0.000	0.006	0.131	0.005	0.001	0.260	0.591	0.134	0.541
**CD163**	0.699	0.762	0.704	0.704	0.000		0.004	0.000	0.000	0.003	0.612	0.009	0.003	0.019	0.981	0.827	0.837
**CD206**	0.186	0.117	0.729	0.729	0.013	0.004		0.001	0.003	0.122	0.853	0.559	0.713	0.001	0.006	0.222	0.207
**CD204**	0.755	0.936	0.880	0.871	0.000	0.000	0.001		0.000	0.295	0.081	0.009	0.099	0.043	0.212	0.399	0.276
**PU1**	0.580	0.900	0.336	0.656	0.000	0.000	0.003	0.000		0.001	0.412	0.000	0.000	0.015	0.236	0.045	0.413
**PD-L1**	0.098	0.344	0.975	0.587	0.006	0.003	0.122	0.295	0.001		0.265	0.006	0.000	0.001	0.086	0.506	0.919
**iNOS**	0.951	0.305	0.106	0.140	0.131	0.612	0.853	0.081	0.412	0.265		0.174	0.312	0.768	0.044	0.212	0.599
**CD3**	0.464	0.500	0.266	0.266	0.005	0.009	0.559	0.009	0.000	0.006	0.174		0.000	0.018	0.614	0.374	0.370
**CD8**	0.492	0.632	0.145	0.714	0.001	0.003	0.713	0.099	0.000	0.000	0.312	0.000		0.129	0.533	0.209	0.774
**FoxP3**	0.053	0.316	0.685	0.265	0.260	0.019	0.001	0.043	0.015	0.001	0.768	0.018	0.129		0.552	0.637	0.740
**Bacterial burden**	0.943	0.342	0.508	0.900	0.591	0.981	0.006	0.212	0.236	0.086	0.044	0.614	0.533	0.552		0.000	0.016
**Bacterial burden (Gram−)**	0.204	0.159	0.057	0.343	0.134	0.827	0.222	0.399	0.045	0.506	0.212	0.374	0.209	0.637	0.000		0.000
**Bacterial burden (Gram+)**	0.933	0.473	0.607	0.652	0.541	0.837	0.207	0.276	0.413	0.919	0.599	0.370	0.774	0.740	0.016	0.000	

Supplementary Figure S1. Prognostic value of bacterial burden/FoxP3 positive cells ratio in ESCC.

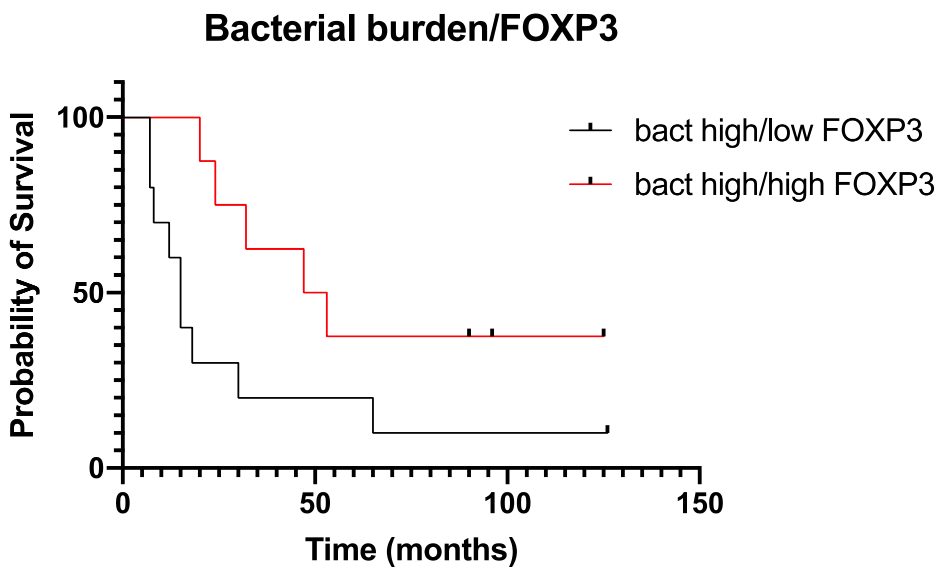

Supplement: Supplementary file 1 [file biomedicines-09-00743-s001.zip › biomedicines-1237048-supplementary.pdf]
